# A Rare Case of Inflammatory Luminal B (HER2-Positive) Breast Cancer Misdiagnosed in a 30-Year-Old Woman: A Case Report

**DOI:** 10.7759/cureus.95528

**Published:** 2025-10-27

**Authors:** Jenny A Zablah, Jorge J Calderon, Angeli N Burgos, Jose A Gasca, Kevin A Montoya, Leiby Orellana Banegas, Marlene Gracia

**Affiliations:** 1 General Practice, Universidad Tecnológica Centroamericana, Tegucigalpa, HND; 2 Internal Medicine, Universidad Nacional Autonoma de Honduras, San Pedro Sula, HND; 3 Emergency Department, Hospital Escuela Universitario, Tegucigalpa, HND; 4 Medicine, Universidad Santiago de Cali, Cali, COL; 5 Health Sciences, Universidad Nacional Autonoma de Honduras, San Pedro Sula, HND; 6 General Practice, Universidad Catolica de Honduras, San Pedro Sula, HND; 7 Medicine, Universidad Autonoma de Nuevo Leon, Monterrey, MEX

**Keywords:** breast imaging, differential diagnosis, granulomatous mastitis, her2-positive, inflammatory breast cancer, invasive ductal carcinoma, misdiagnosis, neoadjuvant therapy

## Abstract

A 30-year-old woman presented with a six-month history of a rapidly enlarging, painful mass in the left breast, accompanied by erythema and intermittent fever. Initial imaging suggested a benign lesion, with differential diagnoses including granulomatous mastitis and phyllodes tumor. She was treated empirically with antibiotics and corticosteroids, but her symptoms worsened. Follow-up imaging revealed further mass enlargement and persistent inflammatory changes. A core needle biopsy confirmed human epidermal growth factor receptor 2 (HER2)-positive, lymphocyte-poor invasive ductal carcinoma, Nottingham Grade III, with a high Ki-67 index and extensive necrosis. Staging studies showed no distant metastases. The patient is recommended to undergo neoadjuvant chemotherapy with HER2 targeted therapy, with plans for radical mastectomy and axillary lymph node dissection. This case underscores the diagnostic challenge of distinguishing inflammatory breast conditions. Inflammatory breast symptoms unresponsive to conservative treatment should prompt early biopsy and multidisciplinary evaluation to avoid delays in diagnosis, especially in young women presenting with atypical features.

## Introduction

Inflammatory breast cancer (IBC) is a rare yet highly aggressive subtype of breast cancer, accounting for only 2-4% of all breast cancer cases in the U.S., yet contributing to 7% of breast cancer-related deaths. Clinically, it is characterized by the rapid onset of inflammatory signs within three to six months, affecting a large portion of the breast and presenting with peau d’orange, a palpable mass, and axillary lymphadenopathy [1].

Granulomatous mastitis (GM) is a benign inflammatory disease that primarily affects women of reproductive age with a history of breastfeeding. Clinically, it presents with inflammatory signs in the breast, the presence of a mass, and, in some cases, unilateral fistulas. In clinical presentation and radiologic appearance, GM often mimics IBC, posing a diagnostic challenge for clinicians [2,3]. Studies have shown that IBC is frequently misdiagnosed as mastitis; one study reported a 38% rate of initial misdiagnosis [4].

On the other hand, phyllodes tumors are rare breast neoplasms originating in the breast’s connective tissue. The majority are benign, with only a small percentage being malignant [5]. A delayed diagnosis can negatively impact the patient’s prognosis or, conversely, lead to unnecessary mastectomies, so it is important to consider all possible diagnostic alternatives when evaluating these conditions [3,6].

We present the case of a 30-year-old woman with no comorbidities or family history of breast cancer who exhibited symptoms and imaging findings suggestive of GM versus a phyllodes tumor. However, histopathologic analysis revealed an inflammatory invasive ductal carcinoma (IDC). This case represents a diagnostic challenge and provides valuable insights.

## Case presentation

A 30-year-old female presented with a progressively enlarging left breast mass over six months, from June 2024 to November 2025, reporting rapid growth throughout this period. She described localized pain and intermittent febrile episodes beginning two months before seeking medical evaluation in November, but denied weight loss, anorexia, or fatigue.

The patient’s gynecological and medical history includes a gravida one, para one, abortion zero, with a fetal demise at term in 2013. Her date of last childbirth was in 2018, denying breastfeeding to any of her children. She experienced menarche at age 14 and has no known chronic illnesses or regular medication use. She also denies smoking history or exposure to toxins. She reported postpartum galactorrhea following the fetal demise. There is no reported family history of breast cancer.

**Figure 1:**
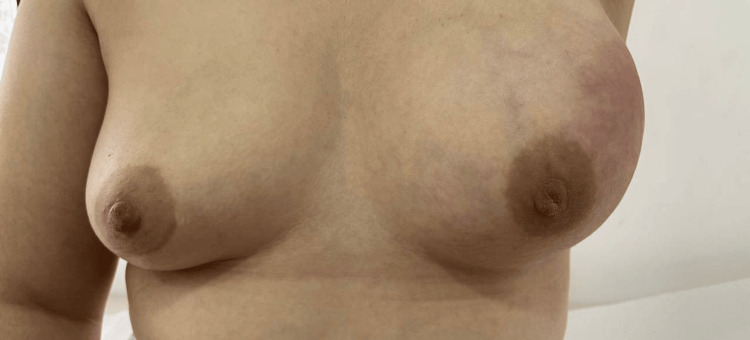
Clinical photograph showing the left breast with erythema of the upper right quadrant and breast enlargement

**Figure 2:**
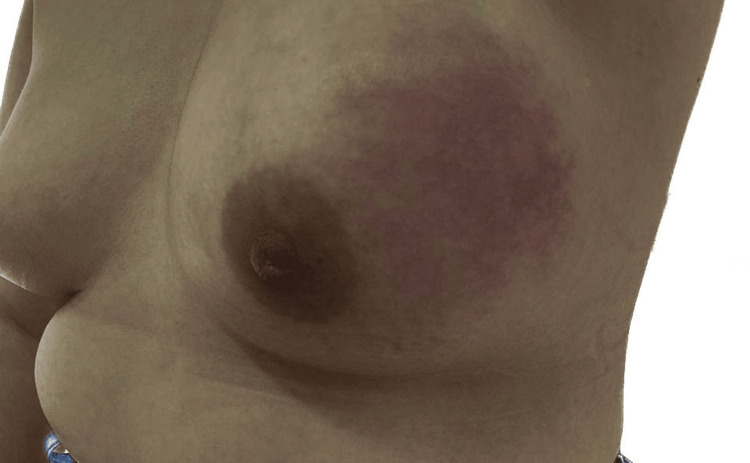
Clinical photograph showing the lateral view of the left breast with erythema of the upper right quadrant and breast enlargement

An initial physical examination revealed asymmetry of the breasts, characterized by the left breast appearing significantly enlarged. A firm, non-mobile mass measuring 8 × 10 cm was palpated in the same quadrant (Clock Position: 12-3, Line B-A). The mass had irregular borders and was tender to palpation. No palpable axillary lymphadenopathy was identified. The right breast presented no abnormalities (Figure 1 and Figure 2).

A prior ambulatory breast ultrasound classified the lesion as Breast Imaging Reporting and Data System (BI-RADS) 4B, describing a large, well-defined, anechoic, oval mass measuring 4.77 × 7.55 × 4.20 cm (79.12 cc), involving all four quadrants of the left breast. A trabecular pattern was noted within the lesion, with preservation of normal breast architecture. Furthermore, a sentinel lymph node was visualized in the left axilla. Due to the limited reliability of the initial ultrasound, a new breast ultrasound was requested, with a follow-up appointment scheduled upon the availability of the results.

**Figure 3:**
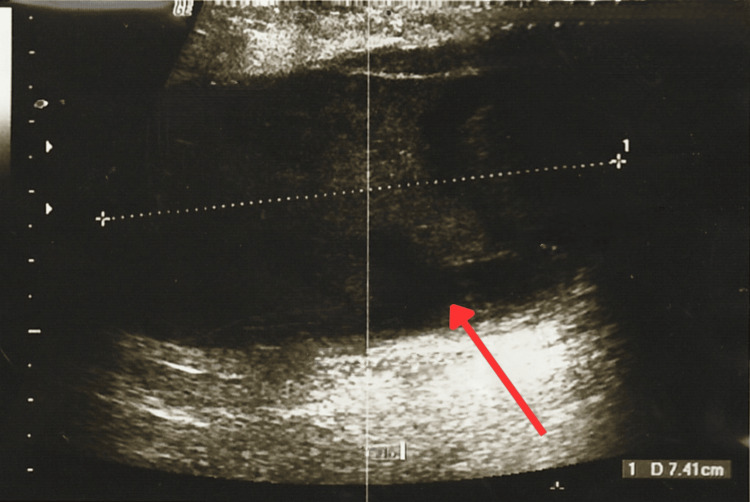
New breast ultrasound where an oval mass with central cystic degeneration can be observed

**Figure 4:**
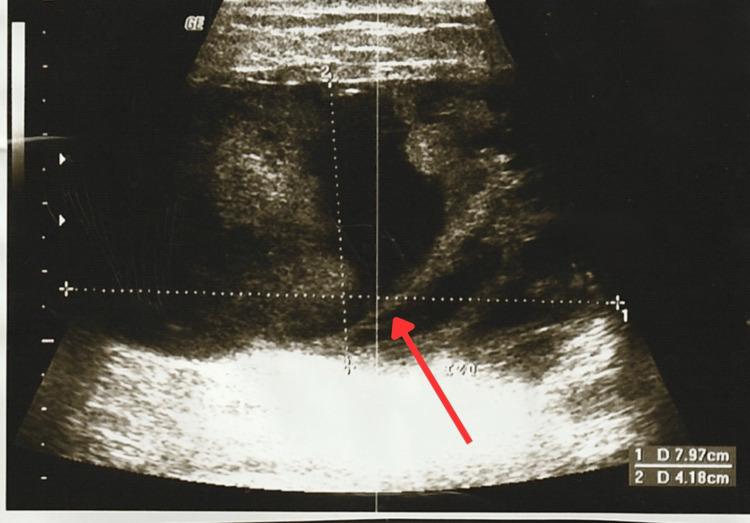
New breast ultrasound where an oval mass with central cystic degeneration can be observed

**Figure 5:**
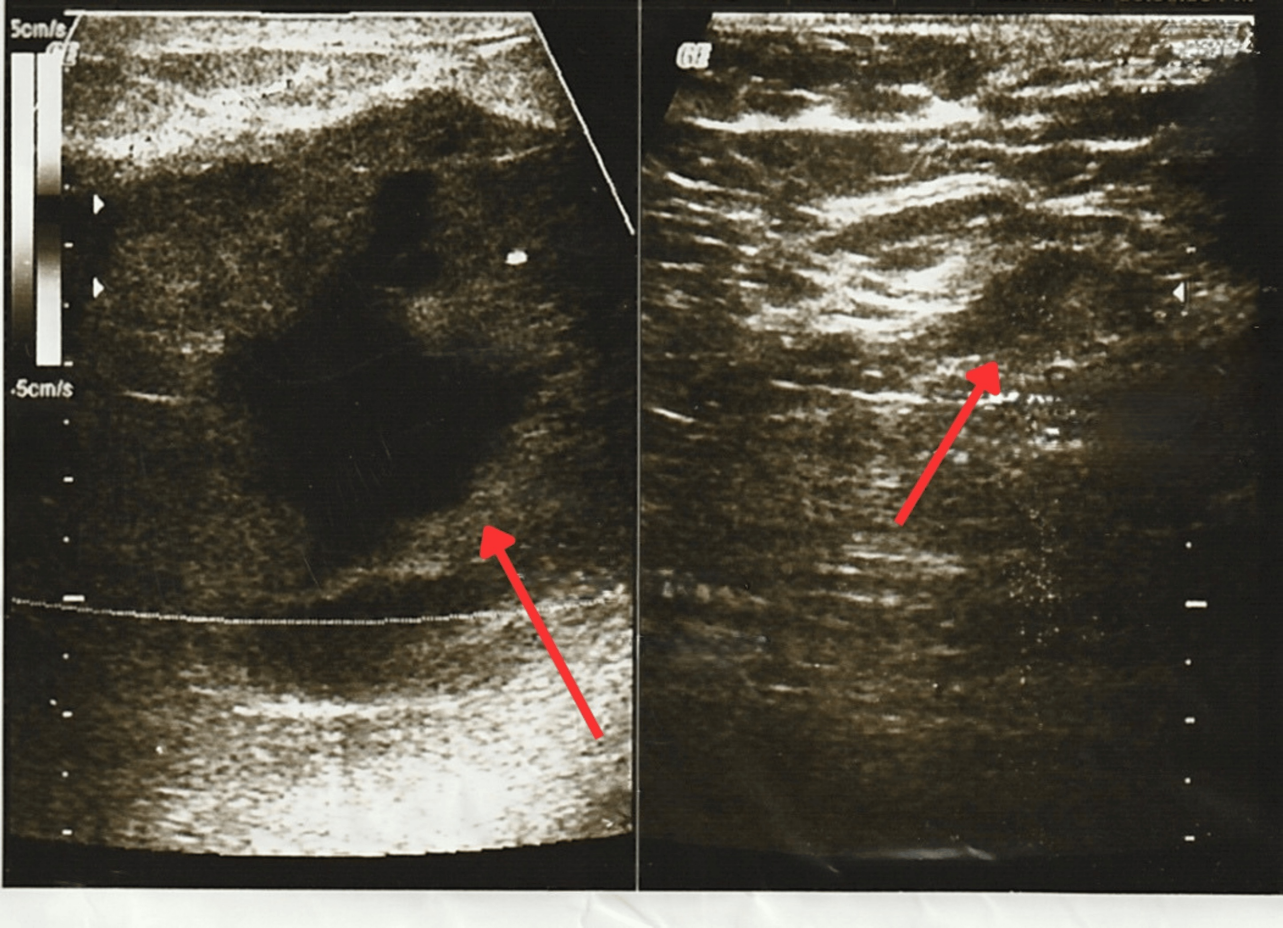
New breast ultrasound where an oval mass with central cystic degeneration and vascularity can be observed First image arrow: Retroareolar mass and vascularity Second image arrow: Oval mass with central cystic degeneration

At the follow-up appointment, the patient reported febrile episodes, redness, warmth, and pain. The physical examination revealed a 10 × 10 cm fixed, firm mass in the upper outer quadrant of the left breast, extending across the intramammary line into the lower outer quadrant and retroareolar region (Clock Position: 12-4, Line B-A). The mass had irregular borders, was tender and warm, and was associated with erythematous skin changes and inflammatory findings. New breast ultrasound classified the lesion as BI-RADS 3, identifying a solid, hypoechoic, well-defined, oval mass with central cystic degeneration and peripheral vascularity on Doppler, measuring 7.9 × 4.1 × 7.4 cm and extending from the upper outer, upper inner, and inner central quadrants to the nipple region. No axillary lymphadenopathy was detected. These findings were interpreted as suggestive of a probable phyllodes tumor (Figure 3, Figure 4 and Figure 5).

Given the clinical findings that initially suggested inflammatory mastitis of the left breast, the patient was prescribed tetracycline 500 mg by mouth (PO) every eight hours for 10 days and deflazacort 15 mg PO daily for two weeks, followed by a tapering regimen. Follow-up imaging was scheduled to monitor treatment response.

**Figure 6:**
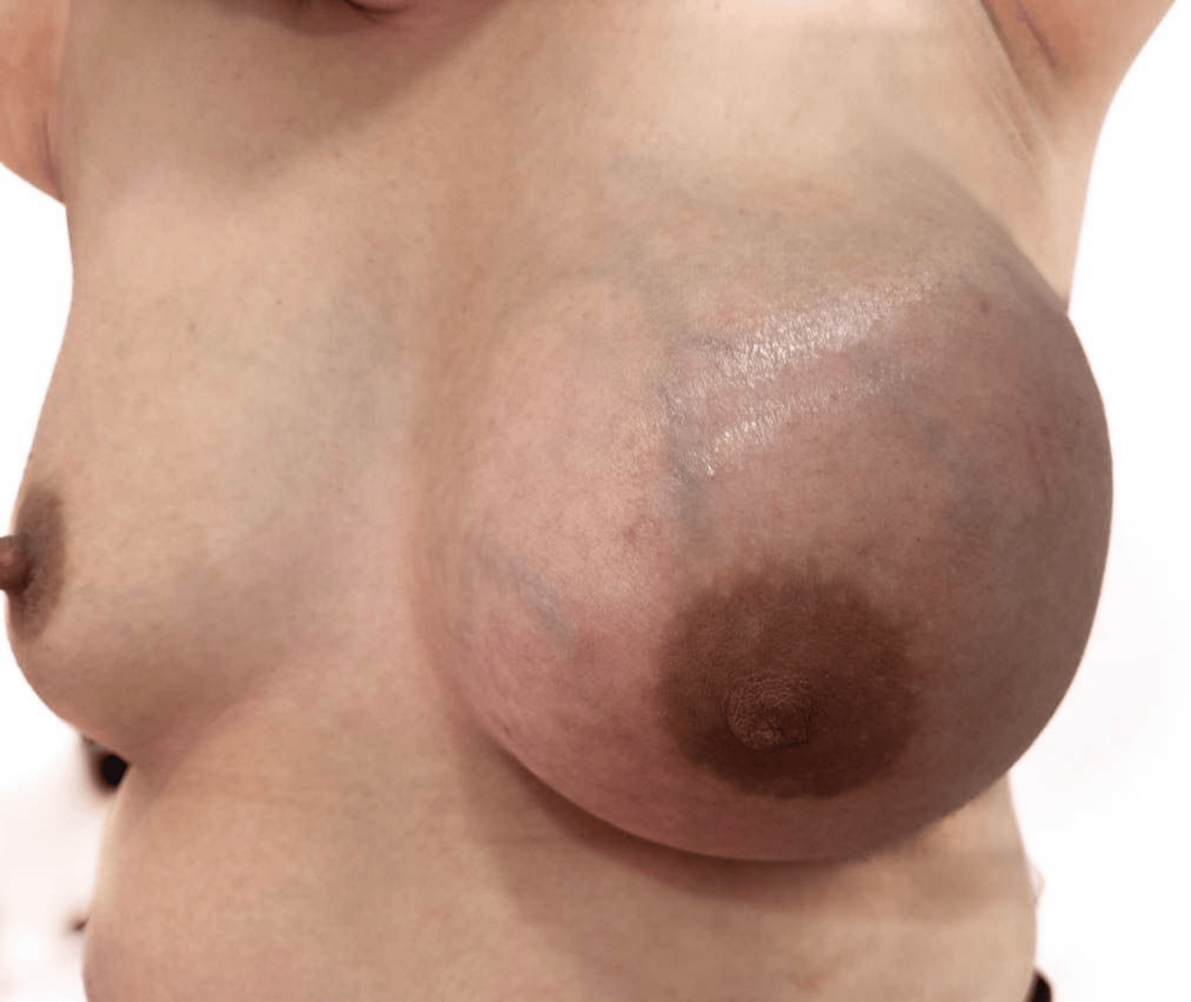
Clinical photograph showing the left breast with diffuse erythema, increased vascularity and breast enlargement

**Figure 7:**
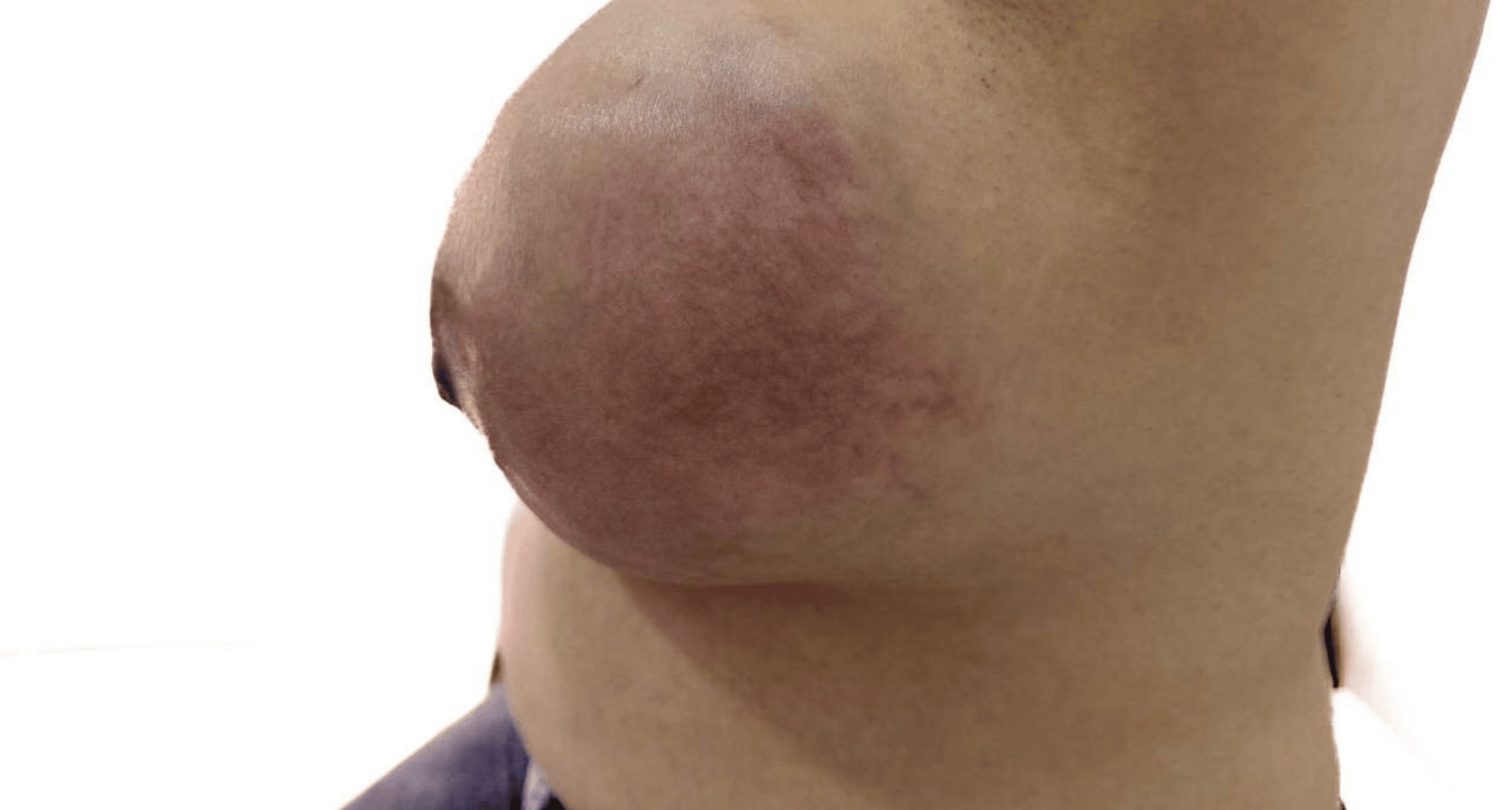
Clinical photograph showing the lateral view of the left breast with diffuse erythema and breast enlargement and nipple retraction

After completing the antibiotic regimen, the patient underwent a follow-up evaluation, which revealed further enlargement of the left breast mass, now extending to involve the entire breast. Inflammatory skin changes and increased vascularity persisted (Figure 6 and Figure 7).

Due to the absence of clinical improvement and continued rapid progression, treatment was adjusted to levofloxacin 750 mg PO daily for seven days and deflazacort 30 mg PO daily with dose escalation. Additionally, laboratory workup and biopsy were requested due to the differential considerations of GM versus phyllodes tumor, particularly in light of the tumor's aggressive growth pattern.

Laboratory work-up revealed notable abnormalities, including an elevated CA 15-3 level (with limited specificity for diagnosis), along with increased aspartate aminotransferase (AST), alanine aminotransferase (ALT), alkaline phosphatase (ALP), gamma-glutamyl transferase (GGT), and lactate dehydrogenase (LDH) (Table 1).

**Table 1:** Laboratory Findings on Evaluation AST: Aspartate Transaminase; ALT: Alanine Transaminase; ALP: Alkaline Phosphatase; GGT: Gamma-Glutamyl Transferase; LDH: Lactate Dehydrogenase

Laboratory Test	Patient Value	Reference Range	Interpretation
CA 15-3	204.61 U/mL	0-30 U/ml	Elevated
AST	49.52 U/L	5-40 U/L	Mildly Elevated
ALT	89.26 U/L	7-56 U/L	Elevated
ALP	210.04 U/L	44-147 U/L	Elevated
GGT	64.50 U/L	9-36 U/L	Elevated
LDH	870.13 U/L	125-220 U/L	Markedly Elevated

**Figure 8:**
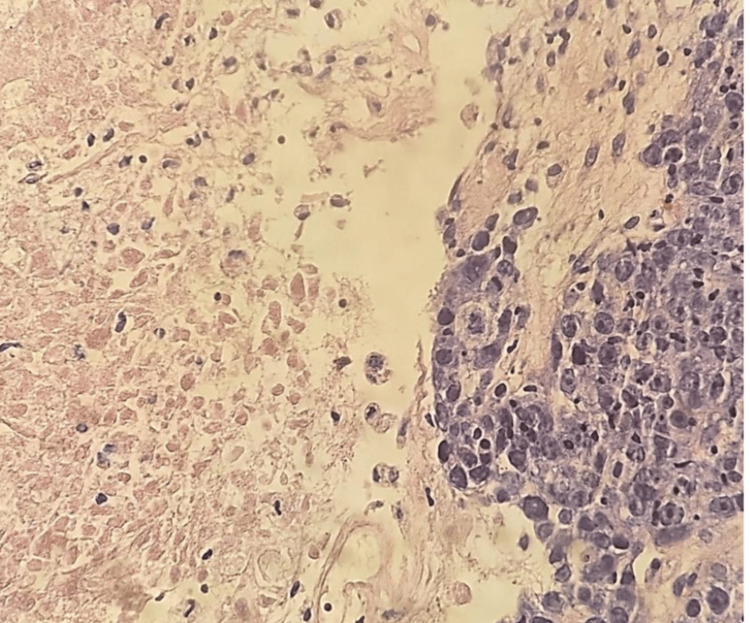
Malignant neoplastic aggregates and necrotic areas

**Figure 9:**
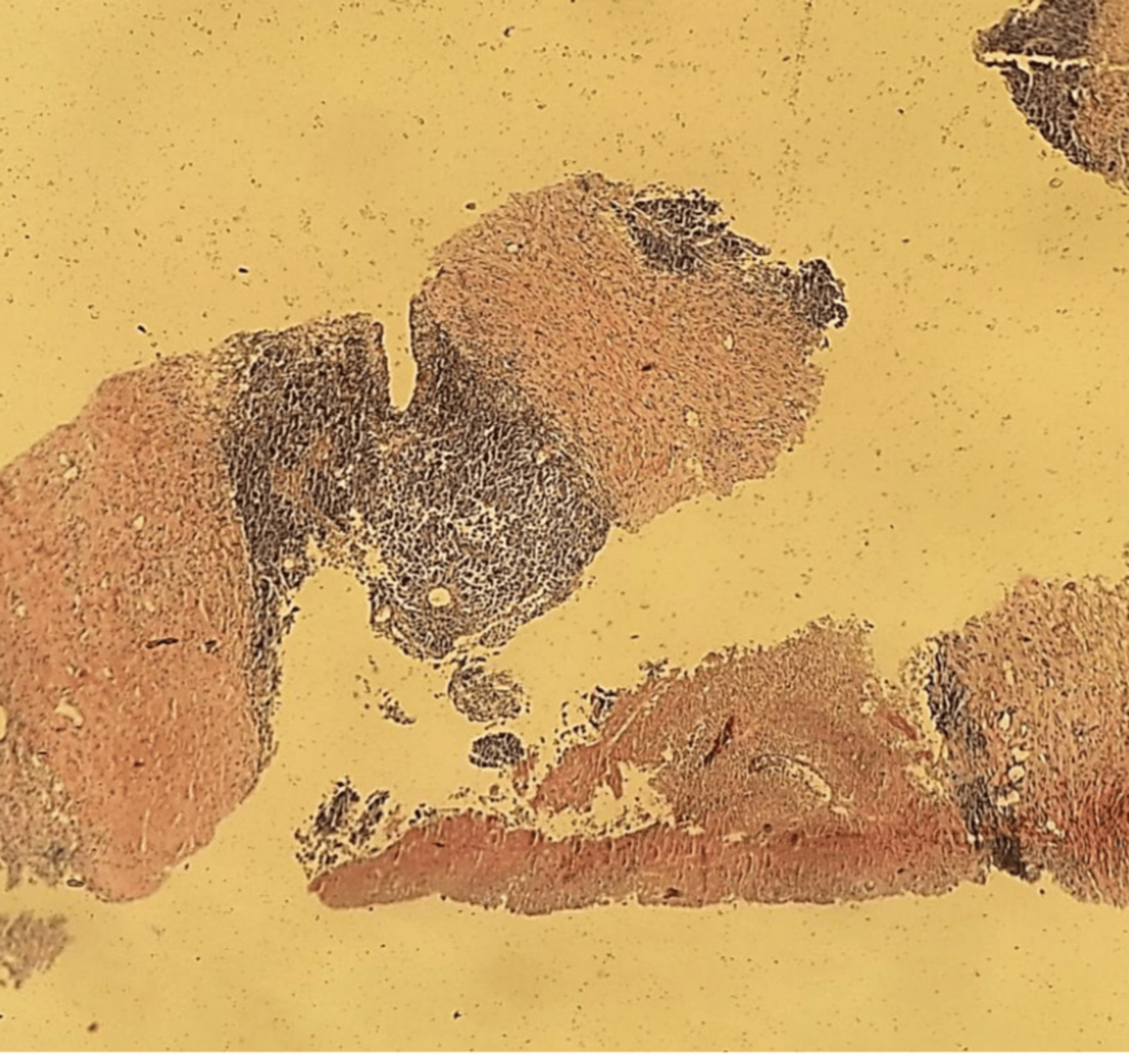
Extensive necrotic areas and foci of fibrosis

**Figure 10:**
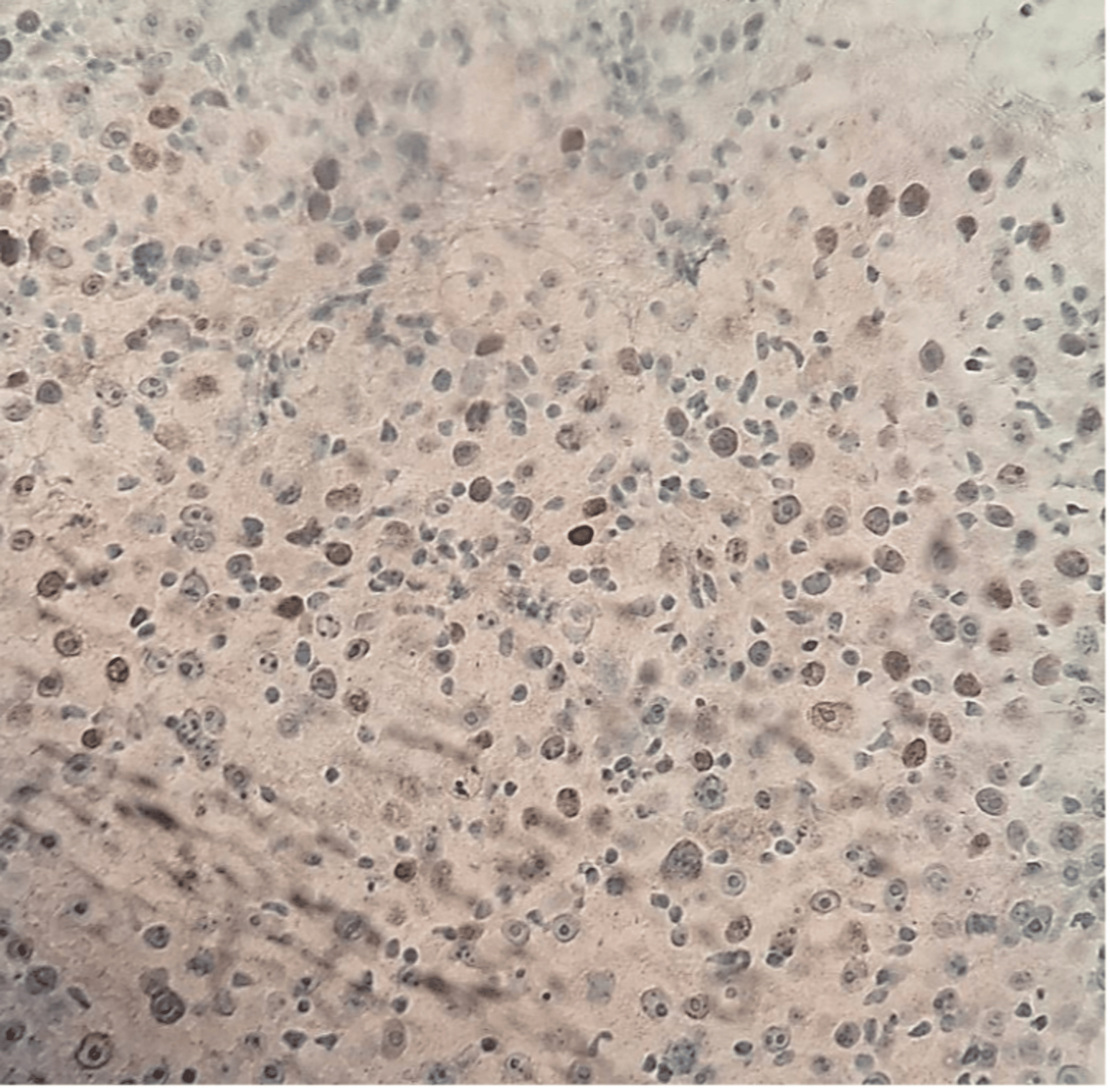
Estrogen receptor: positive (Score 5/8)

**Figure 11:**
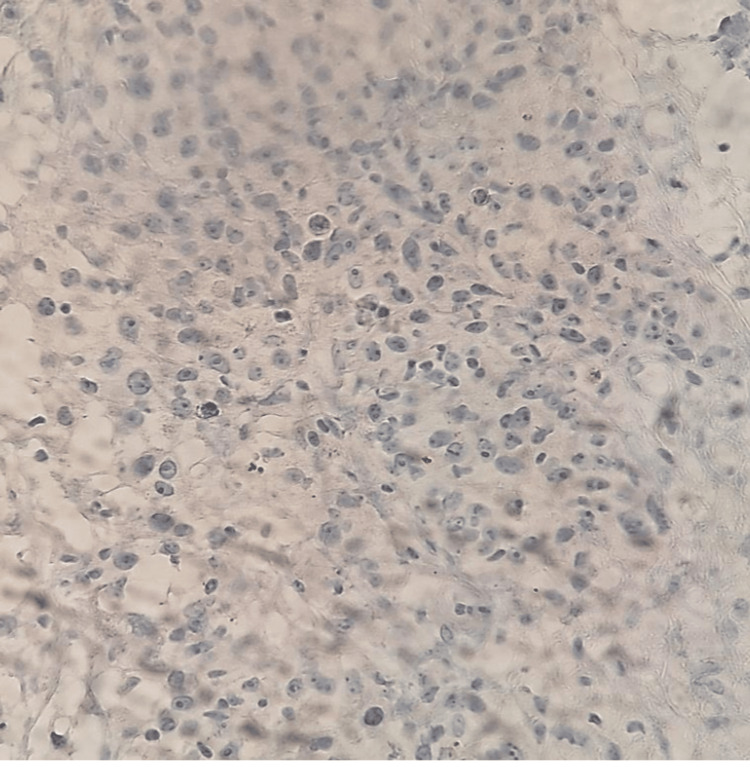
Progesterone receptor: negative (Score 0/8)

**Figure 12:**
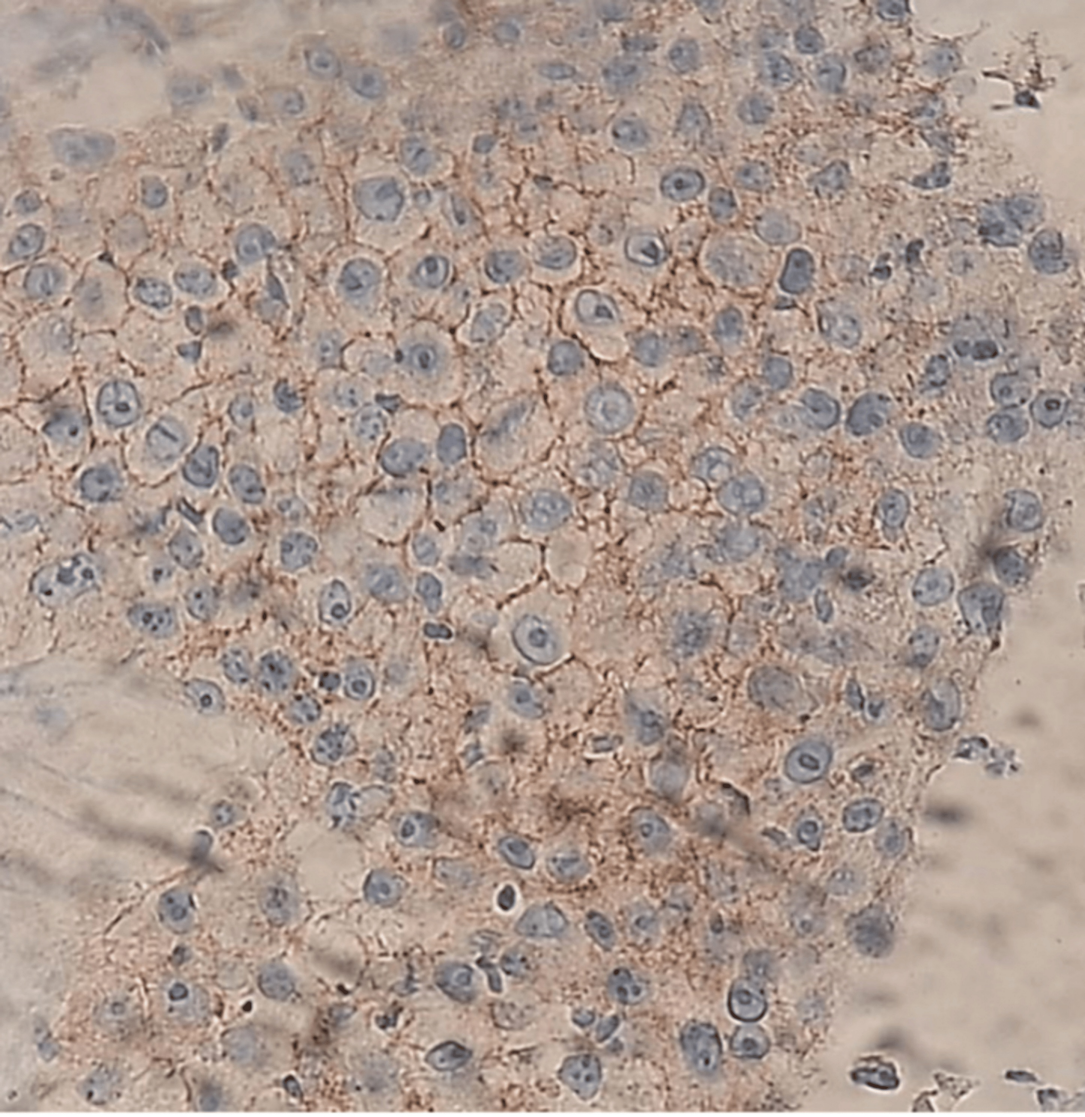
Human epidermal growth factor receptor 2 (HER2) (Neu): positive (Score 3/3)

**Figure 13:**
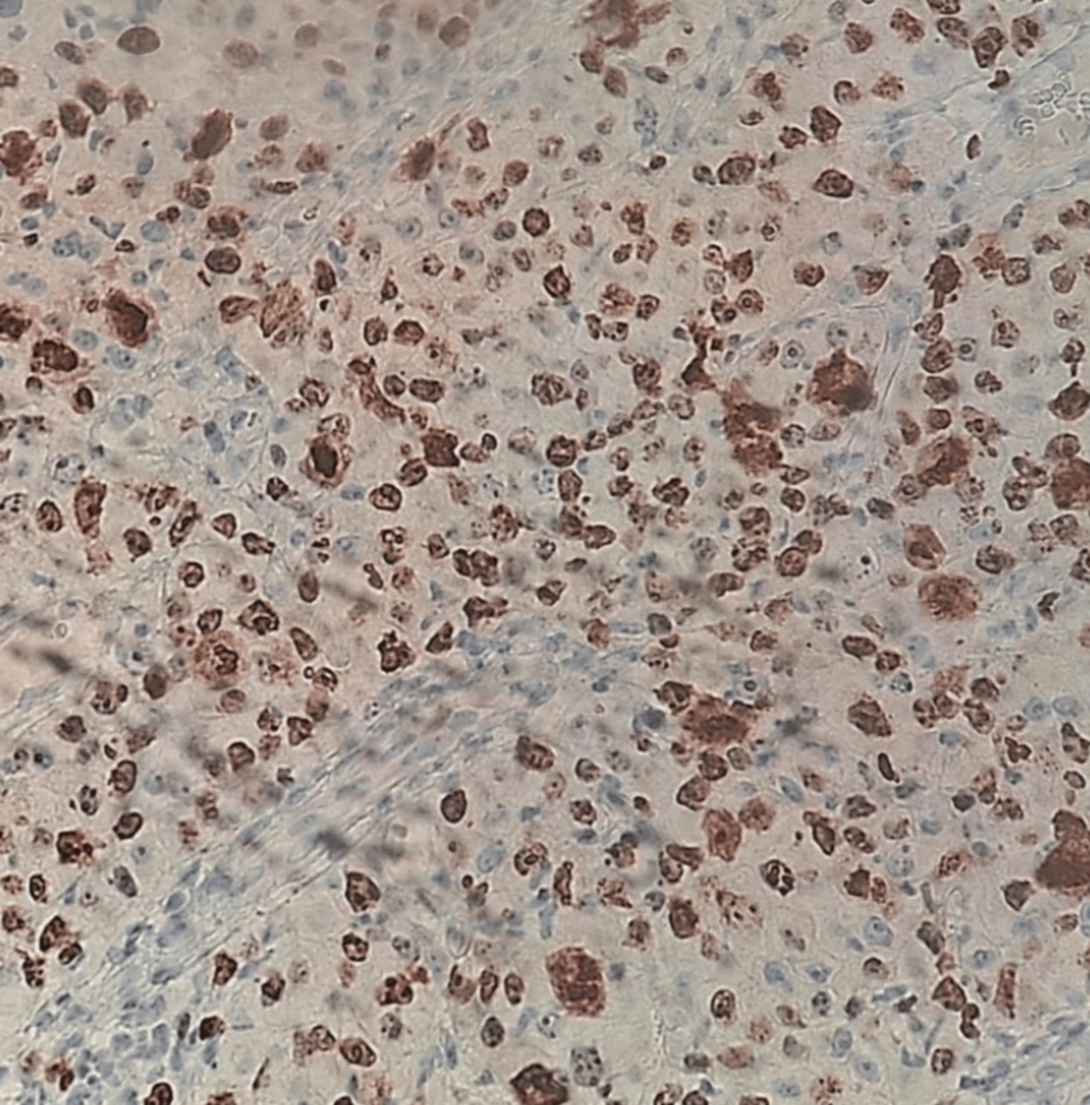
Ki-67: 50%

Given these findings, a core needle biopsy (TRU-CUT) of the nodular lesion was performed, revealing IDC, lymphocyte-poor, Nottingham Grade III, occupying 50% of the sampled tissue with extensive necrosis. No evidence of vascular, lymphatic, or perineural invasion was identified (Figure 8 and Figure 9). Immunohistochemistry demonstrated an estrogen receptor (ER)-positive (Figure 10), progesterone receptor (PR)-negative (Figure 11), human epidermal growth factor receptor 2 (HER2)-positive profile (Figure 12), with a Ki-67 proliferation index of 50% (Figure 13), indicating a high proliferative rate.

**Figure 14:**
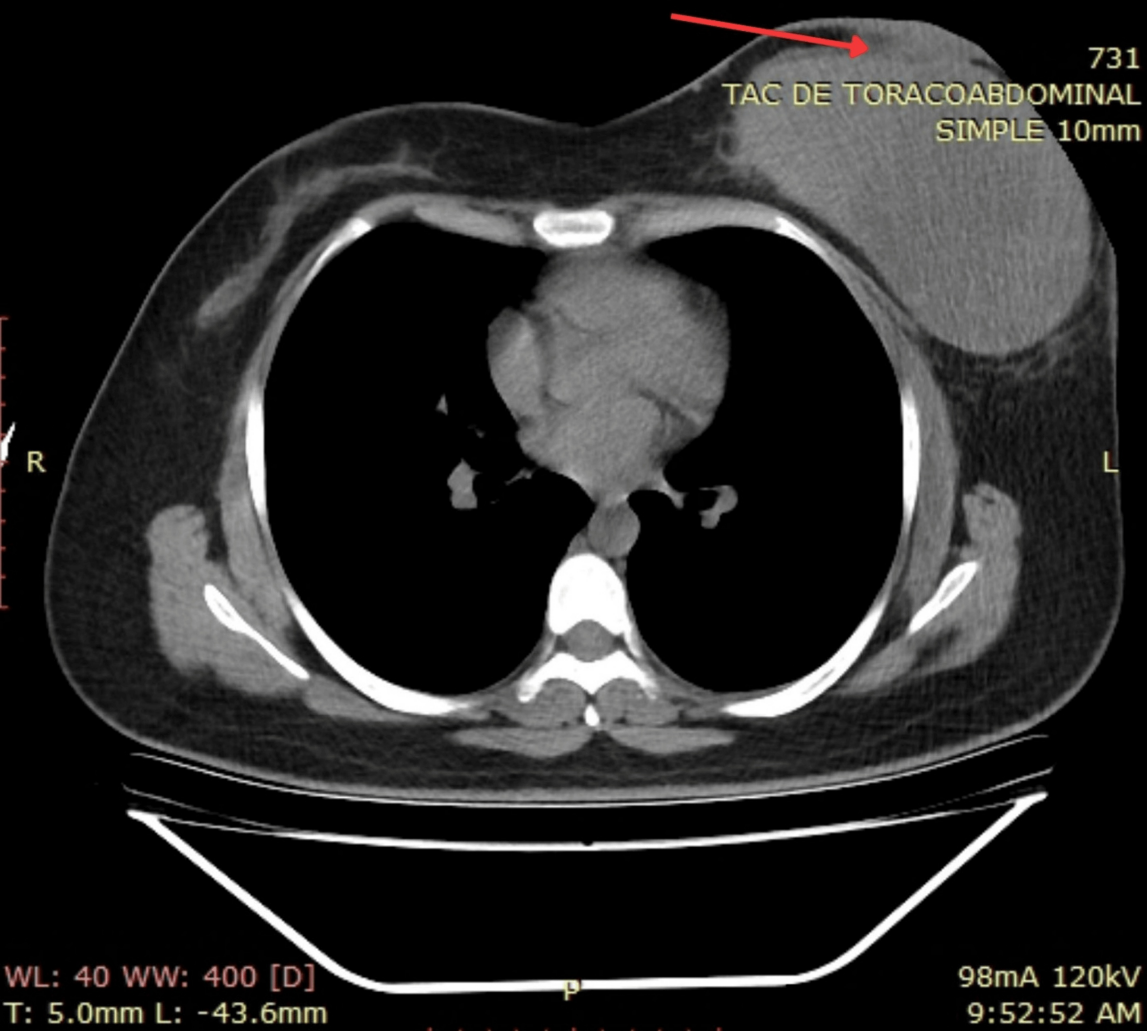
Thoracic CT scan showing a left breast mass, having contact with the skin and loss of the fatty plane

**Figure 15:**
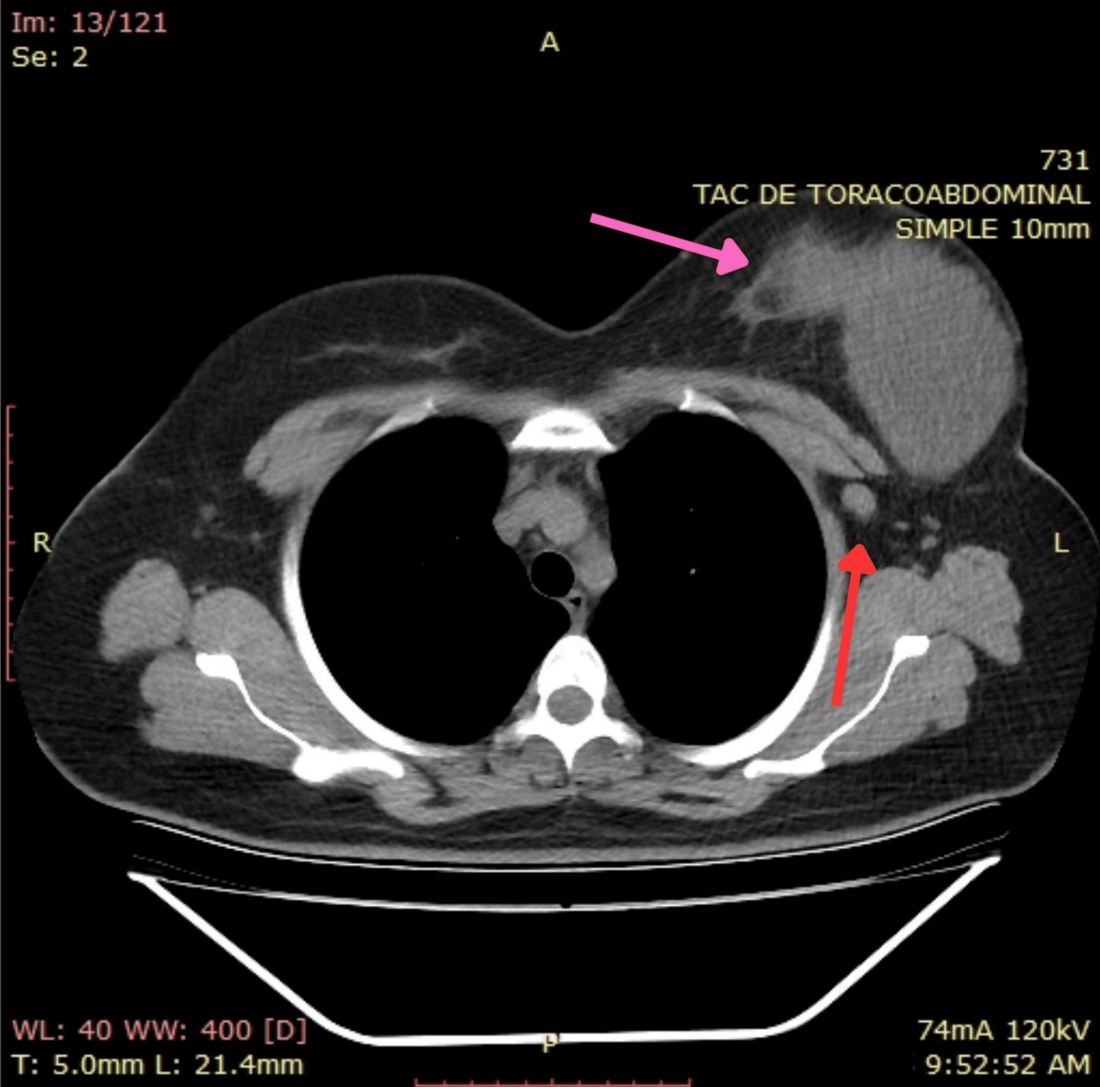
Thoracic CT scan showing a left breast mass, having contact with the skin and ipsilateral extension to the left axillary region Red arrow: Extension to left axillary region Pink arrow: Left breast mass having contact with skin

Subsequent staging imaging was conducted. A thoracic CT scan identified a mixed solid-cystic mass with a predominantly solid peripheral component measuring 13 × 8.5 cm, exhibiting intense contrast enhancement in the solid portion. The lesion demonstrated contact with the skin and loss of the fatty plane, with ipsilateral extension to the left axillary region. An abdominal CT scan showed no evidence of distant metastases (Figure 14 and Figure 15).

Based on the clinical, histopathologic, and radiologic findings, the final diagnosis was locally advanced inflammatory IDC of the left breast, Luminal B (HER2+), with no evidence of distant metastasis. The patient was referred to a higher-level hospital for breast cancer management. The recommended treatment plan involved the initiation of neoadjuvant therapy, including HER2-targeted therapy (trastuzumab) and chemotherapy (carboplatin and docetaxel), followed by surgical evaluation. Given the tumor size and clinical presentation, a total radical mastectomy with axillary lymph node dissection was advised.

## Discussion

Breast cancer in young women is relatively uncommon, comprising fewer than 2% of all cases. Notably, HER2+ tumors are diagnosed in about 26% of these patients [7]. IBC is an aggressive and rare subtype, comprising 1-5% of breast cancers, with a higher incidence in regions such as North Africa, where it may reach up to 10% [8]. It is associated with a poor prognosis and a high risk of early metastatic spread, with 20-40% of cases presenting with distant disease at diagnosis [9]. Epidemiological factors such as obesity, early menarche, and delayed childbirth have been linked to increased risk, particularly in African American populations [8]. Another risk factor that contributes to IBC is smoking [10]. In contrast, our patient lacked these characteristics, reinforcing that IBC can occur even in younger, low-risk women and that reliance on demographic risk factors alone can be misleading.

The diagnostic overlap between IBC and GM is well-established. GM affects women of reproductive age and may present with breast masses, erythema, and pain, often following lactational changes [6,11]. Its clinical similarity to IBC, along with radiologic overlap, can delay appropriate diagnosis. Literature emphasizes that GM often responds to corticosteroids and antibiotics, whereas IBC does not [12]. In this case, the absence of treatment response and continued progression should have raised earlier suspicion for malignancy. This reflects findings that up to 30% of IBC cases are initially misdiagnosed as mastitis, resulting in delayed biopsy and intervention [12]. It also highlights a broader system-level issue: the absence of escalation protocols and over-reliance on benign radiologic interpretations may contribute to delays, particularly in younger patients.

Similarly, phyllodes tumors can resemble IBC in clinical presentation. They often present as rapidly enlarging, firm breast masses that can produce erythema or peau d’orange as seen in this case. In malignant cases, the aggressive growth may mimic IBC with breast enlargement and skin involvement, potentially leading to diagnostic confusion. Therefore, histopathology remains essential for establishing correct diagnosis [5]. Histologically, phyllodes tumors are biphasic fibroepithelial neoplasms characterized by leaf-like (phyllodal) architecture, stromal hypercellularity, and epithelial-lined cleft-like spaces [13]. Grading is based on the degree of stromal cellularity and atypia, mitotic count, stromal overgrowth, and tumor borders, distinguishing benign, borderline, and malignant forms [13].

Radiologic evaluation further complicated the diagnostic course. Initial classification as BI-RADS 4B appropriately suggested malignancy, yet the subsequent downgrade to BI-RADS 3 contributed to false reassurance. This variability illustrates the known limitations of ultrasound and mammography in distinguishing IBC from benign inflammatory disease [14]. While MRI offers superior sensitivity and PET-CT can aid in metastatic assessment [15], histopathology remains the definitive diagnostic method [6]. In this case, biopsy confirmed a high-grade, HER2-positive, lymphocyte-poor IDC with extensive necrosis - findings associated with biologically aggressive tumors [16]. Lymphocyte-poor tumors may demonstrate lower immune infiltration, which could correlate with a poorer prognosis and underscores the urgency of prompt treatment. Although CA 15-3 was elevated, tumor markers remain nonspecific and should never delay histologic confirmation.

Treatment aligned with recommended protocols for HER2-positive IBC, combining neoadjuvant chemotherapy, HER2-targeted therapy, and surgery [15]. The use of anthracycline- and taxane-based regimens has been shown to improve surgical outcomes and survival [15], and the decision for total mastectomy with axillary lymph node dissection was appropriate for locally advanced disease [12]. The multidisciplinary approach employed reflects best practice guidelines, ensuring timely and coordinated care [12]. Despite no evidence of distant metastases in this case, the overall prognosis remains guarded, with five-year survival rates between 25-50% [8]. This case reinforces that persistent inflammatory breast symptoms, even in young patients, warrant early biopsy to avoid treatment delays. It also underscores the importance of clinician education and early multidisciplinary involvement to ensure malignancy is not overlooked when inflammatory symptoms persist.

## Conclusions

This case exemplifies the diagnostic complexity inherent in differentiating IBC from GM, particularly in young women presenting with rapidly progressive breast masses. The initial clinical and radiological overlap with benign inflammatory conditions can delay oncologic intervention and negatively impact prognosis. In this patient, early misclassification on imaging and empiric antibiotic treatment highlighted the limitations of relying solely on imaging and clinical response to guide diagnosis. Histopathological analysis remains the gold standard, and its timely implementation is essential when patients do not respond to initial therapy.

Despite ongoing advances in imaging and molecular profiling, the etiology and early detection of inflammatory breast cancer remain poorly understood, particularly in young women. Further multicenter and population-based research is needed to clarify risk factors, molecular drivers, and the role of breastfeeding and hormonal influences in pathogenesis. From a system-level standpoint, especially in regions with limited access to advanced imaging, implementing standardized protocols that mandate early biopsy for non-resolving mastitis in young women could significantly improve diagnostic timelines and reduce treatment delays. The identification of HER2-positive IDC with inflammatory features underscores the need for a high index of suspicion for malignancy when breast inflammation persists or worsens, even if imaging initially suggests a benign process. This case reinforces the value of a multidisciplinary approach that integrates imaging, pathology, and clinical evaluation to ensure timely diagnosis and treatment planning. In HER2-positive IBC, early initiation of targeted therapy and chemotherapy significantly improves response rates and outcomes. Ultimately, any rapidly enlarging, non-resolving inflammatory breast lesion should prompt early biopsy to avoid delays that may compromise the window for curative treatment and surgical intervention.
